# The Role of the Atherogenic Index of Plasma and the Castelli Risk Index I and II in Cardiovascular Disease

**DOI:** 10.7759/cureus.74644

**Published:** 2024-11-28

**Authors:** Isha Raaj, Manvitha Thalamati, Vanitha Gowda M N, Akshay Rao

**Affiliations:** 1 Department of Biochemistry, M. S. Ramaiah Medical College, Bengaluru, IND; 2 Department of Internal Medicine, M. S. Ramaiah Medical College, Bengaluru, IND

**Keywords:** abdominal obesity, asian indian, atherogenic index of plasma, body mass index: bmi, cardiovascular disease, castelli risk index, insulin resistance, metabolic syndrome, obesity, visceral fat

## Abstract

Introduction: Metabolic syndrome (MS), identified by abdominal obesity, insulin resistance, hypertension, and/or dyslipidemia, occurs across all BMI (body mass index) ranges and increases the risk of atherosclerotic cardiovascular (CV) diseases and type II diabetes. The Atherogenic Index of Plasma (AIP) and Castelli Risk Index (CRI) I & II are ratios that can be calculated from a simple lipid profile test. These ratios are independent risk factors for CV diseases and have been shown to be increased in angiographically confirmed coronary artery disease (CAD) patients. This study aimed to assess CV risk across the different subtypes of obesity: metabolically obese non-obese (MONO), metabolically healthy non-obese (MHNO), metabolically obese obese (MOO), and metabolically healthy obese (MHO) using AIP and CRI I & II and to study the association of AIP, CRI I & II with other CV risk factors such as total body fat percentage (BF%), visceral fat percentage (VF%), and BMI. Assessing CV risk in an individual based on the person's subtype of obesity using ratios calculated from simple lipid profile parameters may prove beneficial to developing better screening strategies.

Methods: A cross-sectional study was conducted on 128 adults with BMI ≥18.5 kg/m^2^ with and without MS, presenting to the General Medicine/Internal Medicine Outpatient Department in M S Ramaiah Medical College Hospital, Bangalore, Karnataka State, India. The sample size was calculated to be a minimum of 82 subjects based on a study that showed that AIP and CRI I & II had a positive association with BMI. After a detailed history, physical examination, anthropometric measurements (height, weight, and waist circumference), VF%, and BF% by bio-impedance were recorded. A blood sample was processed for lipid profile and fasting blood sugar on a Vitros 5600 auto-analyzer (Quidel Corporation and Ortho Clinical Diagnostics, San Diego, CA, USA). Subjects were divided into MONO (non-obese subjects with BMI < 25 kg/m^2^ having MS), MHNO (no obesity or MS), MOO (obese BMI ≥ 25 kg/m^2^ having MS), and MHO (obese BMI ≥ 25 kg/m^2^ not having MS) groups. AIP and CRI I & II were calculated. Statistical analysis was performed using the chi-square test, ANOVA, Pearson correlation coefficient, and receiver operating characteristic curve (ROC).

Results: MONO, MHNO, MOO, and MHO constituted 26 (20.3%), 48 (37.5%), 28 (21.8%), and 26 (20.3%) of the 128 subjects, respectively. AIP ≥0.24 was found in 16 (61.5%) of MONO and in 16 (51.1%) of MOO subjects. CRI-I >4 was found in 19 (73.1%) and 16 (57.1%) subjects of the MONO and MOO groups, respectively. Eleven (42.3%) and 12 (42.9%) of MONO and MOO subjects, respectively, had CRI-II >3. Pearson's correlation revealed for AIP r=0.32, p=0.000 and r=0.43, p=0.000 with VF% and BMI, respectively. The area under the curve (AUC) for AIP and CRI I & II to detect the presence of MS were 0.84, 0.74, and 0.73, respectively.

Conclusion: CV risk, as assessed by AIP and CRI I & II in the different subtypes of obesity, was found to be highest in the MONO group, followed by the MOO group. With BMI and VF%, AIP showed a moderately positive linear correlation. API and CRI could be tools of low cost and moderate reliability in screening the general population for risk of CV disease.

## Introduction

Obesity, defined by excessive fat accumulation, poses significant health risks and has reached epidemic levels globally. It is a major contributor to various health conditions, including cardiovascular (CV) diseases, stroke, type 2 diabetes, infertility, polycystic ovary syndrome, musculoskeletal disorders, and certain cancers [1]. Interestingly, not all individuals with obesity experience the same health risks, and some with a normal body mass index (BMI) may still have an elevated CV risk. To address this, different subtypes of obesity have been identified, such as MONO (metabolically obese non-obese or non-obese subjects with BMI < 25 kg/m^2^ having metabolic syndrome (MS)), MHNO (metabolically healthy non-obese or no obesity or MS), MOO (metabolically obese obese or obese with BMI ≥ 25 kg/m^2^ having MS), and MHO (metabolically healthy obese or obese with BMI ≥ 25 kg/m^2^ not having MS) groups [2].

In a study by Geetha et al., the prevalence of coronary artery disease in MONO, MOO, MHO, and MHNO was found to be 5.5%, 4.2%, 1.4%, and 2.6%, respectively [2]. Both the MONO and MOO groups are associated with MS, a cluster of conditions including abdominal obesity, insulin resistance, hyperglycemia, hypertriglyceridemia, hypertension, and decreased HDL cholesterol [2]. Most MONO individuals are likely undetected for CV risk because of normal BMI. Considering the intricacies of metabolic health in obesity and the prevalence of CV disease (CVD), reliable markers are needed to predict CV risk among these individuals, which would help in developing better screening strategies for early detection and treatment of CVD, especially in MONO individuals.

The Castelli Risk Index (CRI) I & II and the Atherogenic Index of Plasma (AIP) are simple ratios that can be calculated from the lipid profile values of a subject and have been used as screening tools to identify increased CV risk [3,4]. CRI-I measures the ratio of total cholesterol (TC) to HDL (high-density lipoprotein) cholesterol, while CRI-II is based on the ratio of LDL (low-density lipoprotein) to HDL cholesterol. A higher CRI-I indicates an increased risk of developing CVD because TC includes both the LDL and other lipid components, while HDL is considered the "good" cholesterol, which is a lipoprotein that helps in cholesterol excretion through reverse cholesterol transport. An elevated TC/HDL-C ratio indicates low HDL-C levels relative to the TC, increasing the risk of arterial atherosclerotic plaque formation (atherosclerosis), which can lead to heart attacks. A value of CRI-I >4 is considered an indicator of increased risk of CVD and reflects the formation of coronary plaques. A high CRI-II of >3 predicts an increased risk of CVD, acute myocardial infarction, and insulin resistance.

Conversely, AIP is a logarithmic ratio of triglycerides (TG) to HDL cholesterol and indicates high CV risk. AIP has been shown to be high when CV risk parameters such as TG and HDL cholesterol are normal. A higher AIP is associated with MS, obesity, and insulin resistance and suggests a higher risk of CVD due to atherosclerosis. An AIP of -0.3 to 0.11 indicates a low risk of CVD, 0.11-0.24 indicates an intermediate risk of CVD, and ≥0.24 indicates a high risk of CVD [3,4].

BMI, visceral fat percentage (VF%), and total body fat percentage (BF%) are also factors that influence CV risk. Visceral fat releases pro-inflammatory cytokines and free fatty acids that contribute to insulin resistance, abnormal lipid metabolism, elevated BP, type 2 diabetes, and CVD. It is a critical risk factor for the development of metabolic abnormalities associated with MS. Excess body fat and high BMI are considered to be critical contributors to the development and progression of MS [1,2]. Thus, it is also important to study the association of these factors with AIP and CRI I & II.

This study aimed to assess CV risk using AIP and CRI I & II across the different subtypes of obesity (MHNO, MHO, MONO, and MOO, classified based on BMI and the presence or absence of MS) and to study the association of AIP and CRI I & II with BF%, VF%, and BMI.

## Materials and methods

Study design and setting

This cross-sectional study was conducted at the General Medicine/Internal Medicine Outpatient Department of the M. S. Ramaiah Medical College Hospital, Bangalore, Karnataka State, India.

Ethical considerations

This study obtained ethical clearance from the Institutional Ethics Committee of Ramaiah Medical College (Ethics Committee Reg. No. ECR/215/Inst/KA/2013/RR-22, Protocol EC No. MSRMC/EC/PG-1/07-2022).

Sampling/rationale for sample size

From the literature review, a study by Bhardwaj et al. reported that AIP and CRI I & II had positive associations with BMI (r=0.22, p=0.008) and CV risk [4]. In the present study, expecting a similar result with 80% power and a 95% confidence level and to get a minimum correlation coefficient of 0.5, the study required a minimum of 82 subjects. The G*Power software (Heinrich Heine University Düsseldorf, Düsseldorf, Germany) was used for sample size calculation. The total sample size (N) was calculated using the formula [(Zα+Zβ)/C]2 + 3, where Zα is the standard normal deviate for α, Zβ is the standard normal deviate for β, r is the correlation coefficient, and C = 0.5 * ln[(1+r)/(1-r)].

Sampling

It was decided to include the first 128 subjects consecutively who fulfilled the study's inclusion criteria. After calculating BMI, measuring BP, and obtaining results of lipid profile and fasting blood sugar (FBS), these 128 subjects were categorized into MHNO, MOO, MONO, and MHO [4].

Data collection

A non-probability sample from attendees to the Internal Medicine Department was selected according to certain inclusion criteria, namely, age ≥18 years with BMI ≥18.5 kg/m^2^ over the period from October 2022 to December 2022. The following were the exclusion criteria for the study: subjects with a history of abnormal thyroid function test (serum TSH (thyroid-stimulating hormone) levels above or below the reference range 0.4-4.5 µU/mL), liver function test (abnormal serum total bilirubin, aspartate transaminase, alanine transaminase, alkaline phosphatase, gamma-glutamyl transferase, and albumin), and renal function test (eGFR (estimated glomerular filtration rate) calculated by CKD-EPI Creatinine Equation < 90 mL/minute/1.73 m^2^) [5,6]. Subjects on treatment during the past six months for any thyroid, liver, and renal-related disorders, undergoing dialysis, active cancer treatment (anthracyclines (doxorubicin, etc.), taxanes (paclitaxel), tyrosine kinase inhibitors (imatinib, nilotinib, etc.), rapamycin, alkylating agents (cyclophosphamide, etc.), anti-metabolites (5-fluorouracil, methotrexate, etc.), hormones, and their receptor modulators (estrogen, testosterone, tamoxifen)) were excluded [7]. Women who were pregnant or had delivered less than six months ago were also excluded. Subjects with a history of usage of steroids and statins in the past six months with implanted cardiac pacemakers and/or other implanted electronic devices (contraindicated, as the bioimpedance analyzer (BIA) may interfere with the electromagnetic impulses generated by these implanted devices and leads to malfunction) were excluded from the study.

Written informed consent was obtained from each subject who fulfilled the inclusion criteria and agreed to participate in the study. After a detailed history and physical examination, anthropometric measurements (height, weight, and waist circumference (WC)) were taken. Anthropometric measurements and BP were obtained using standardized techniques and instruments. The instruments (weighing scale, stadiometer, BP apparatus) were calibrated before the data collection.

Subjects were asked to remove hair ornaments and headdresses and stand upright without shoes with their backs against the height-measuring vertical scale of the stadiometer. The back of the head, back, buttocks, calves, and heels were ensured to touch the stadiometer, and the feet were together. The top of the external auditory meatus (ear canal) should be level with the inferior margin of the bony orbit (cheekbone) and eyes directed forward. The height was measured to the nearest centimeter.

Weight (in kilograms) was measured with an electronic scale kept on a firm horizontal surface. Subjects were asked to wear light clothing and stand upright without shoes, and their weight was recorded to the nearest 0.5 kg. BMI was calculated using the following formula: weight (kg)/height (m)^2^.

WC was measured using a non-stretchable fiber measuring tape at the smallest horizontal girth between the costal margins and the iliac crests at the end of expiration directly over the skin. Measurements were taken to the nearest 0.1 cm. For WC, two measurements were made, and the mean of the two readings was taken as the final value.

BP was recorded in the sitting position in the right arm to the nearest 1 mmHg using the mercury sphygmomanometer. Two readings were taken five minutes apart, and the mean of the two was taken as the BP. If the difference between the first and second readings was greater than 10 mmHg for systolic pressure and/or greater than 6 mmHg for diastolic pressure, then a third measurement was made, and the mean of all three measurements was taken as the BP [2].

Body composition was assessed using a bioimpedance body composition analyzer (GOQii Body Composition Monitor, GOQii Technologies, California, USA). This analyzer measured body weight and total body water and subsequently estimated the BF% and VF%.

After an overnight fast of 8-12 hours, approximately 5 mL of blood was drawn with due aseptic precautions in plain vacutainer tubes from each subject included in the study. Then, the samples were allowed to stand until they clot. After centrifugation and serum separation, the serum samples were processed for FBS and lipid profile as a part of the standard of care protocol. The tests on the samples were estimation of TG, TC, LDL, HDL, and VLDL (very-low-density lipoprotein) by enzymatic methods and FBS by the hexokinase method. The autoanalyzer used was a Vitros 5600 analyzer (Quidel Corporation and Ortho Clinical Diagnostics, San Diego, CA, USA).

The indices AIP=log (TG/HDL-C), CRI-I=TC/HDL, and CRI-II=LDL/HDL were calculated [3,4]. An AIP of -0.3-0.11 indicated a low risk of CVD, 0.11-0.24 indicated an intermediate risk of CVD, and ≥0.24 indicated a high risk of CVD. A value of CRI-I >4 was considered an indicator of increased risk of CV disease and reflects the formation of coronary plaques. A value of CRI-II >3 predicts an increased risk of CV disease, acute myocardial infarction, and insulin resistance [8]. Subjects with BMI ≥18.5 kg/m^2^ included in the study were grouped after taking into consideration WC, BP recording, serum TG, HDL, and FBS as MHNO with BMI 18.5-24.9 kg/m^2^ and absence of MS, MONO with BMI 18.5-24.9 kg/m^2^ and presence of MS, MOO with BMI ≥25 kg/m^2^ and presence of MS, and MHO with BMI ≥25 kg/m^2^ and absence of MS.

A diagnosis of MS was made based on laboratory investigations and anthropometric measurements according to the International Diabetes Federation (IDF) and the National Cholesterol Education Program Adult Treatment Panel III (NCEP ATP III) for the South Asian population. The presence of any three of the following five modifiable CV risk factors made a clinical diagnosis of MS. The five factors are elevated WC of ≥90 cm in men and ≥80 cm in women, elevated serum TG of ≥150 mg/dL, reduced serum HDL of <40 mg/dL in males and <50 mg/dL in females, elevated BP of ≥130 mmHg systolic blood pressure (SBP) and/or ≥85 mmHg diastolic blood pressure (DBP) or on antihypertensive drug treatment in a patient with a history of hypertension, elevated FBS of ≥100 mg/dL or on drug treatment for elevated glucose [2].

Data analysis

Data analysis was conducted using statistical tools, specifically MS Excel (Microsoft Corporation, Redmond, Washington) and IBM SPSS Statistics for Windows, Version 22 (Released 2013; IBM Corp., Armonk, New York). The chi-square test or Fischer's exact test was used as a significance test for qualitative data. ANOVA was used as a significance test to identify the mean difference between more than two quantitative variables. The correlations were performed with the Pearson correlation coefficient. Receiver operating characteristic curves (ROCs) were constructed for the CRI, AIP, and MS. A test that predicts an outcome no better than chance has an area under the ROC curve of 0.5. An area under the ROC curve above 0.8 indicated a fairly good prediction. A P-value (probability that the result is true) of <0.05 was considered statistically significant after assuming all the rules of statistical tests.

## Results

This study included 128 subjects (65 female and 63 male subjects, who fulfilled the inclusion criteria). The subjects were categorized into the following groups based on BMI and the presence or absence of MS: MHNO with 37.5% (48), MHO with 20.3% (26), MONO with 20.3% (26), and MOO with 21.8% (28). The male patients accounted for 41.6% (20) in MHNO, 38.46% (10) in MHO, 57.6% (15) in MONO, and 64.28% (18) in MOO (Table 1). The females accounted for 58.3% (28) in MHNO, 61.5% (16) in MHO, 42.3% (11) in MONO, and 35.7% (10) in MOO (Table 1). The distribution of males was found to be the highest in the MOO group, followed by the MONO group. The distribution was found to be highest among females in the MHO, followed by the MHNO group. The oldest age distribution was found in the MONO group (51.5±11.1), and the youngest was in the MHNO group (27.3±11.9).

**Table 1:** Demographic characteristics of the study population. MHNO: metabolically healthy non-obese; MHO: metabolically healthy obese; MONO: metabolically obese non-obese; MOO: metabolically obese obese

Demographic Characteristics	MHNO	MHO	MONO	MOO
Percentage (number) n=128	37.5%(48)	20.3%(26)	20.3%(26)	21.8%(28)
Age (mean±SD) in years	27.3±11.9	42.3±13.4	50.1±7.2	51.5±11.1
Gender % (number) male n=63; female, n=65	41.6%(20); 58.3%(28)	38.46%(10); 61.5%(16)	57.6%(15); 42.3%(11)	64.28%(18); 35.7%(10)

AIP and CRI I & II were highest in the MONO and MOO groups, as shown in Table 2. With a P-value of 0.005, the VF% showed a significant difference with all four subtypes of obesity. With a P-value of 0.000, the AIP and CRI I & II showed a significant difference among all four subtypes of obesity.

**Table 2:** The association of total body fat%, visceral body fat%, BMI, and waist/hip ratio with the CRI I & II and AIP in various obesity subtypes. BMI: body mass index; AIP: Atherogenic Index of Plasma; CRI-I: Castelli Risk Index I; CRI-II: Castelli Risk Index II; MHNO: metabolically healthy non-obese; MHO: metabolically healthy obese; MONO: metabolically obese non-obese; MOO: metabolically obese obese

Parameters	MHNO (n=48)		MHO (n=26)		MONO (n=26)		MOO (n=28)		P-value
	Mean	SD	Mean	SD	Mean	SD	Mean	SD	
BMI	21.70	2.35	28.72	3.82	23.73	1.01	30.36	5.16	0.001
Waist/hip ratio	0.90	0.07	0.90	0.05	0.90	0.04	0.92	0.05	0.609
Body fat %	24.57	8.21	35.56	7.58	28.03	7.81	34.52	7.49	0.065
Visceral fat %	3.96	2.69	12.22	6.38	12.98	3.50	13.69	3.91	0.005
AIP=log (TG/HDL-C)	-0.12	0.23	-0.02	0.22	0.27	0.26	0.26	0.27	0.000
Castelli index I = TC/HDL	3.55	0.92	3.76	0.92	4.84	1.48	4.71	1.39	0.000
Castelli index II = LDL/HDL	2.00	0.65	2.26	0.77	2.95	1.04	2.80	1.10	0.000

As shown in Table 3, the distribution of the low-risk group calculated by AIP was found to be the highest (81.3%, 39) in the MHNO group, followed by 80.8% (21) in the MHO group. The intermediate risk of AIP was found to be highest in the MONO group (19.2%, 5), and the least was found to be in the MHO group (12.5%, 3). The highest risk was found to be in the MONO group, with 61.5% (16), followed by 57.1% (16) in the MOO group. With a P-value of <0.001, the AIP showed a significant association with all four subtypes of obesity. CRI-I with a cutoff value of >4 (indicating high CV risk) was found to be highest in the MONO group, with 73.1% (19), followed by 57.1% (16) in the MOO group. With a P-value of 0.006, the CRI-I showed a significant association with all four subtypes of obesity. CRI-II with a cutoff value of >3 (indicating high CV risk) was found to be highest in the MOO group with 42.9% (12), followed by 42.3 (11) in the MONO group. With a P-value of <0.001, the CRI-II showed a significant association with all four subtypes of obesity (Table 3).

**Table 3:** Relationship of cardiovascular risk in subtypes of obesity using AIP and CRI I & II. AIP: Atherogenic Index of Plasma; CRI: Castelli Risk Index; MHNO: metabolically healthy non-obese; MHO: metabolically healthy obese; MONO: metabolically obese non-obese; MOO: metabolically obese obese

Calculated Indexes	MHNO (n=48)		MHO (n=26)		MONO (n=26)		MOO (n=28)		P-value
	N	%	N	%	N	%	N	%	
AIP									
Low cardiovascular risk (-0.3 to 0.11)	39	81.3%	21	80.8%	5	19.2%	8	28.6%	<0.001
Intermediate cardiovascular risk (0.11 to -0.24)	6	12.5%	3	11.5%	5	19.2%	4	14.3%	
High cardiovascular risk (≥0.24)	3	6.3%	2	7.7%	16	61.5%	16	57.1%	
CRI-I									
<4	31	64.6%	17	65.4%	7	26.9%	12	42.9%	0.006
>4 (increased cardiovascular risk)	17	35.4%	9	34.6%	19	73.1%	16	57.1%	
CRI-II									
<3	44	91.7%	22	84.6%	15	57.7%	16	57.1%	0.001
>3 (increased cardiovascular risk)	4	8.3%	4	15.4%	11	42.3%	12	42.9%	

The correlation analysis of BMI, waist/hip ratio, blood sugar, BF%, and VF% with AIP and CRI I & II was conducted using Pearson correlation coefficient (r) among all the study subjects, as shown in Table 4. The BMI, waist/hip ratio, blood sugar, BF%, and VF% exhibited a positive correlation with AIP and CRI I & II. The BMI (r = 0.317), blood sugar (r = 0.403), and VF% (r = 0.429) showed a moderate correlation with the AIP. The waist/hip ratio (r = 0.016) and BF% (0.208) exhibited a weak correlation with the AIP. The BMI (r = 0.231), blood sugar (r = 0.124), waist/hip ratio (r = 0.030), BF% (0.070), and VF% (r = 0.232) showed a weak correlation with the CRI-I. The BMI (r = 0.257) and VF% (r = 0.249) exhibited a moderate correlation with the CRI-II. The waist/hip ratio (r = 0.037), blood sugar (r = 0.074), and BF% (0.069) showed a weak correlation with the CRI-II. With a P-value of <0.05, BMI, blood sugar, BF%, and VF% exhibited a significant association with the AIP. With a P-value of <0.05, BMI and VF% had a significant association with the CRI I & II.

**Table 4:** Correlations were performed using the Pearson correlation coefficient. BMI: body mass index; AIP: Atherogenic Index of Plasma; CRI: Castelli Risk Index

Measured and Calculated Parameters	Statistical Analytical Method	AIP	CRI-I	CRI-II
BMI	Pearson correlation (r)	0.317	0.231	0.257
	P-value	0.000	0.009	0.003
Waist/hip ratio	Pearson correlation (r)	0.016	0.030	0.037
	P-value	0.857	0.738	0.678
Blood sugar mg/dL	Pearson correlation (r)	0.403	0.124	0.074
	P-value	0.000	0.167	0.409
Body fat %	Pearson correlation (r)	0.208	0.070	0.069
	P-value	0.019	0.436	0.442
Visceral fat %	Pearson correlation (r)	0.429	0.232	0.249
	P-value	0.000	0.008	0.004

**Figure 1:**
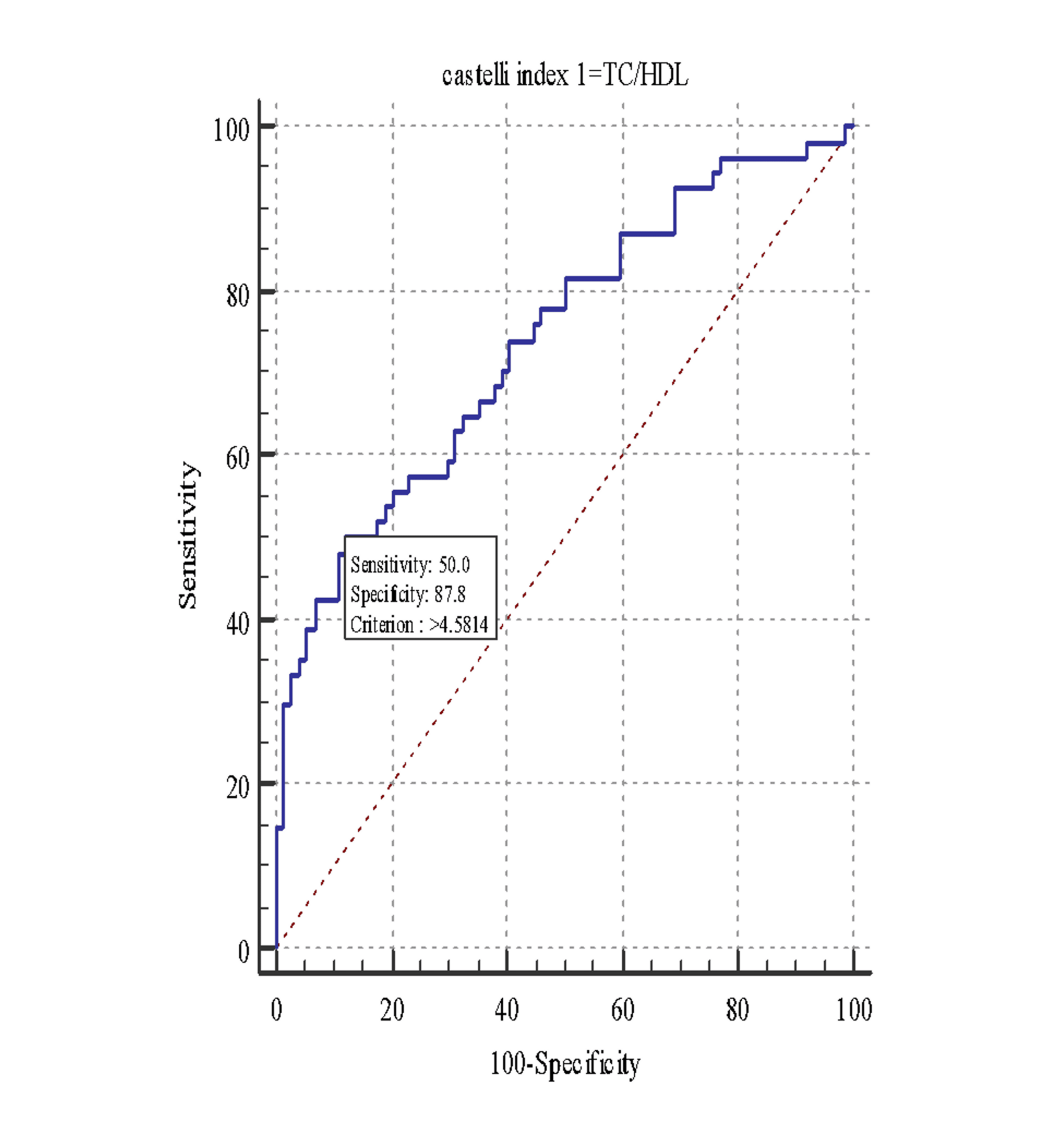
Receiver operating characteristic curve (ROC) for Castelli Risk Index I (CRI-I) and metabolic syndrome. TC/HDL: total cholesterol/high-density lipoprotein

ROC was constructed for CRI I & II, AIP, and MS. With a cutoff of >4.58, the CRI-I to identify the subjects with MS showed an area under the curve (AUC) of 0.74, sensitivity of 50.00, and specificity of 87.84, with a positive predictive value of 75.00 and a negative predictive value of 70.70 (Figure 1).

**Figure 2:**
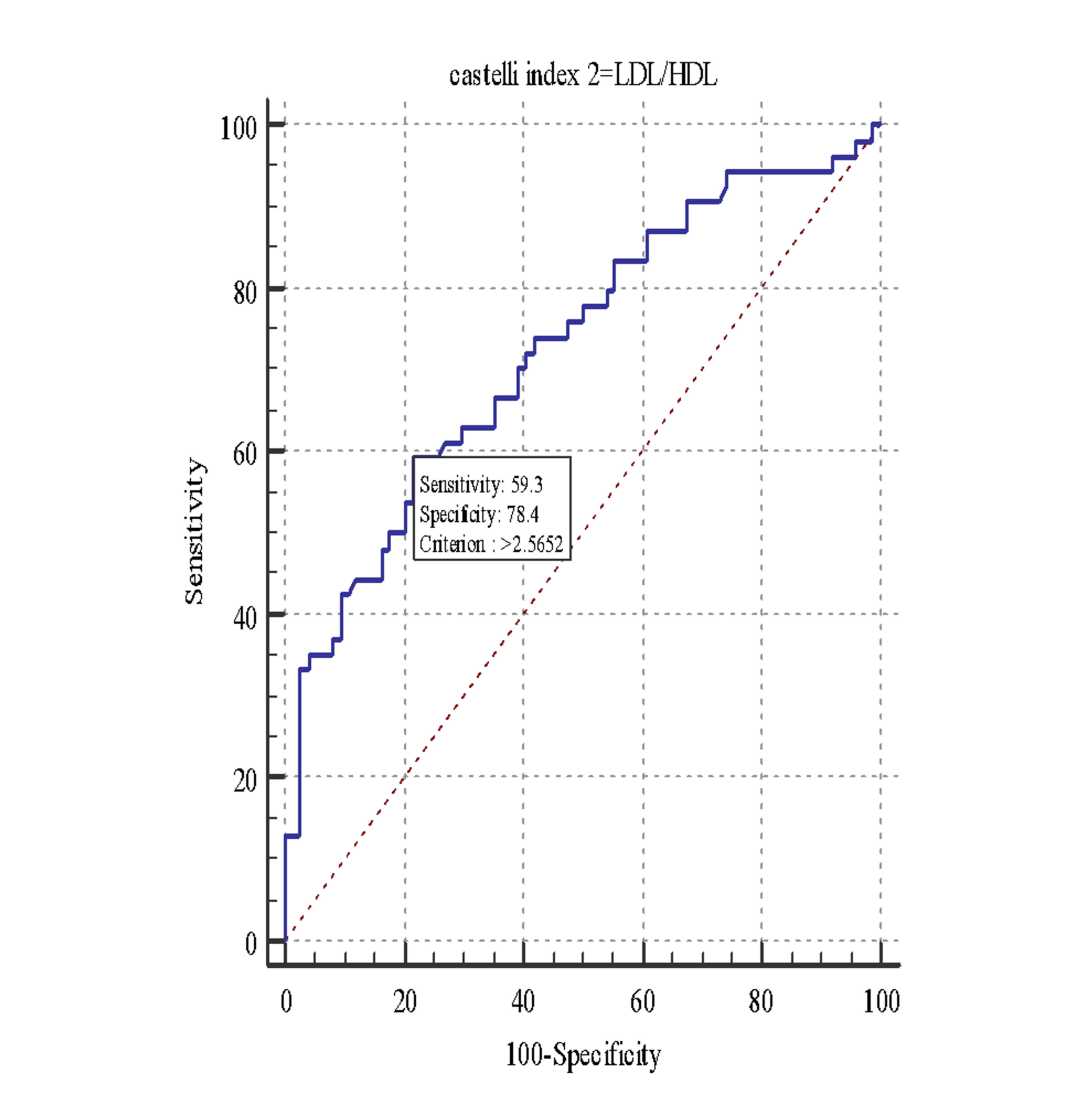
Receiver operating characteristic curve (ROC) for Castelli Risk Index II (CRI-II) and metabolic syndrome. LDL/HDL: low-density lipoprotein/high-density lipoprotein

With a cutoff of >2.26, the CRI-II to identify the subjects with MS showed an AUC of 0.73, sensitivity of 66.67, and specificity of 60.81, with a positive predictive value of 55.40 and a negative predictive value of 71.40 (Figure 2).

**Figure 3:**
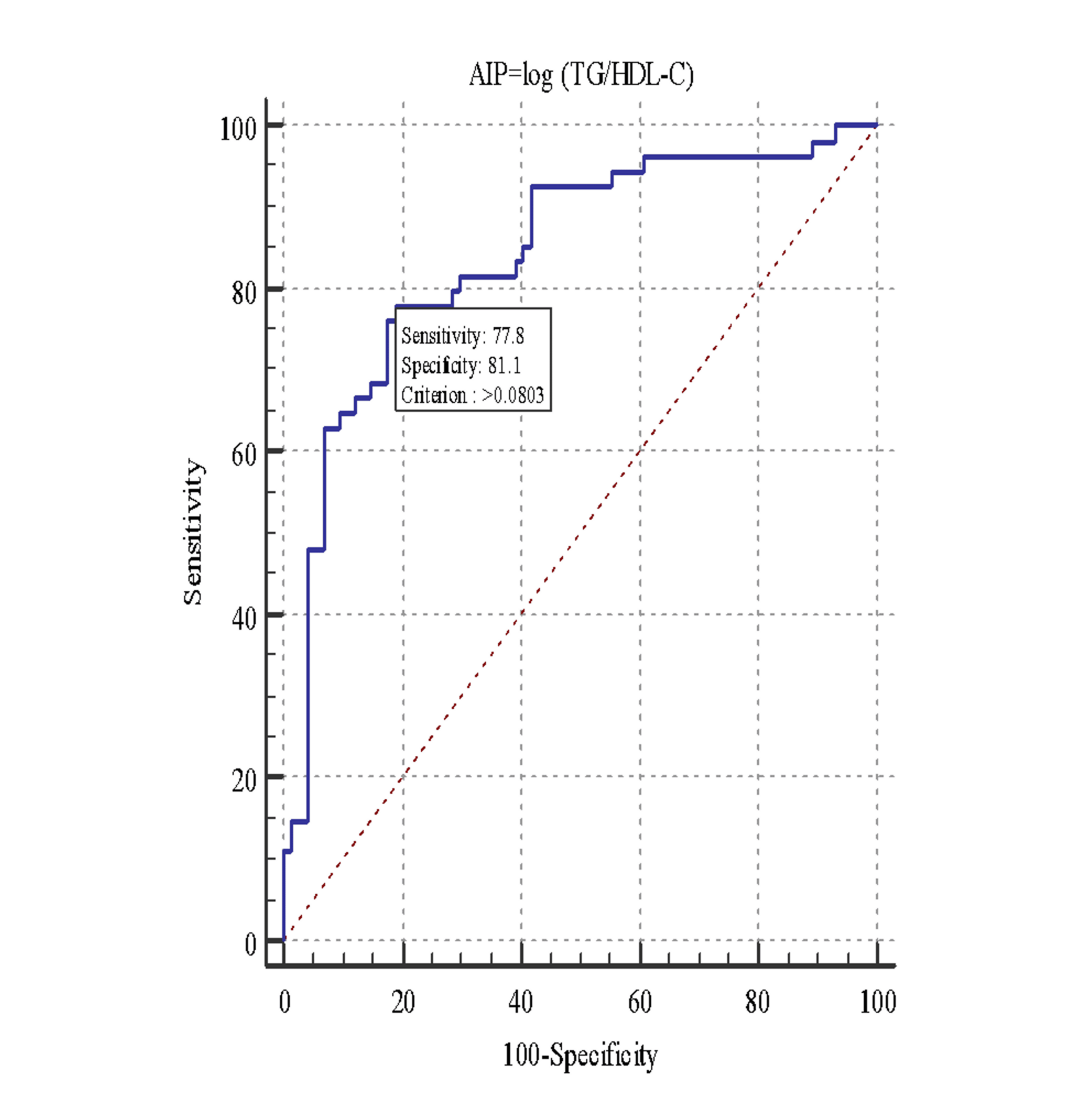
Receiver operating characteristic curve (ROC) for Atherogenic Index of Plasma (AIP) and metabolic syndrome. TG/HDL-C: triglyceride/high-density lipoprotein cholesterol

As shown in Figure 3, with a cutoff of >0.08, the AIP to identify the subjects with MS showed an AUC of 0.84, sensitivity of 77.78, and specificity of 81.08, with a positive predictive value of 75.00 and a negative predictive value of 83.30. As AUC above 0.80 indicated fairly good prediction, AIP (AUC=0.84) had a good prediction capacity for detecting MS when compared with CRI I & II (Table 5).

**Table 5:** ROC curve analysis for CRI-I, CRI-II, and AIP to identify metabolic syndrome. ROC: receiver operating characteristic curve; AIP: Atherogenic Index of Plasma; CRI: Castelli Risk Index

Test Result Variable(s)	CRI-I	CRI-II	AIP
Area under the curve (AUC)	0.74	0.73	0.84
Sensitivity%	50.00	66.67	77.78
Specificity%	87.84	60.81	81.08
+PV	75.00	55.40	75.00
-PV	70.70	71.40	83.30
Cutoff	>4.58	>2.26	>0.08
Significance level P (area=0.5)	<0.0001	<0.0001	<0.0001

## Discussion

The present study was a cross-sectional study that included 128 subjects who were distributed into four subtypes of obesity based on BMI and the presence of MS as follows: MONO (nonobese subjects with BMI < 25 kg/m^2^ having MS), MHNO (no obesity or MS), MOO (obese BMI ≥ 25 kg/m^2^ having MS), and MHO (obese BMI ≥ 25 kg/m^2^ not having MS). MONO constituted 26 (20.3%), MHNO 48 (37.5%), MOO 28 (21.8%), and MHO 26 (20.3%) of the subjects.

In a study by Geetha et al. on an urban population in Chennai, South India, MONO constituted 15.1%, MHNO 56.8%, MOO 14.8%, and MHO 13.3% of the subjects, respectively [2]. The study was a population-based study, unlike the present study, where subjects who visited the hospital for a routine health checkup were included. In the present study, the male gender was predominant in the MOO and MONO groups, and the female gender was predominant in the MHO and the MHNO groups. The MONO group had an older age distribution (mean age in years ± SD of 51.5±11.1), and the youngest age group was found in the MHNO group (mean age 27.3±11.9). In the study by Geetha et al., the MONO group had subjects with a higher mean age, and the lowest mean age group was found in the MHNO group [2].

The assessment of the CV risk using AIP and CRI I & II across these different subtypes of obesity revealed that in the MONO group, AIP and CRI I & II were the highest, having mean±SD values of 0.271±0.2, 4.84±1.4, and 2.95±1.04, respectively. The MOO group also had similar mean±SD values of 0.264±0.27, 4.71±1.3, and 2.80±1.10 for AIP and CRI I & II, respectively. These findings are similar to other studies done in India [4,8-10].

Among the subtypes of obesity, the subjects of MOO had the highest values for BMI, waist/hip ratio, WC, VF%, and FBS with a mean ±SD of 30.36±5.16, 0.92±0.05, 96.32±9.2 cm, 13.69±3.91%, and 133±40 mg/dL, respectively. AIP, calculated as the logarithmically transformed ratio of plasma TG to HDL-C, correlates closely with the LDL particle size (an indicator of the atherogenic lipoprotein phenotype) and predicts CV risk, rendering it a useful measure of the response to treatment.

A study by Shilpa Bhardwaj et al. found that an AIP value of ≥0.24 indicated a high risk of CVD. The study showed that AIP contributes the most among the three indices, approximately 30% to the risk of developing CVD [4]. In the present study, a value of AIP indicating high risk was found maximally in subjects of MONO (61.5%) and MOO (57.1%). Of the three indices, AIP showed a specificity of 81.08% and a sensitivity of 77.78% at a cutoff of >0.08 (AUC of 0.841) to identify subjects of MOO and MONO from the other subtypes of obesity or those having MS. Thus, metabolically obese patients were more prone to develop CVDs irrespective of the BMI.

In a study by Barua et al., BMI was suggested as the strongest predictor of AIP compared with other anthropometric measurements [10]. In the present study, VF% exhibited more strength of association than BMI with AIP. Hence, VF% strongly correlates with CVD risk than anthropometric measurements. A study by Koleva et al. investigated the role of AIP and CRI I & II in MS. CRI-I >4 was found to be significantly higher in subjects with MS. In the present study, CRI-I >4 was significantly higher in the MONO (73.1%) and MOO (57.1%) groups. Koleva et al. found that CRI-II >3 was higher in individuals with MS than in healthy subjects. In the present study, CRI-II >3 was highest in the MOO (42.9%) and MONO (42.3%) groups [11]. AIP≥0.24, CRI-I>4, and CRI-II>3 in this present study in metabolically obese patients are in concordance with other studies. Essiarab et al. studied the impact of obesity and MS on lipoprotein profiles and CV risk in Moroccan women and concluded that MOO subjects exhibited increased AIP values and higher predictive values [12].

It is known that prediction of CV risk based on conventional lipid profile parameters may not be sufficient. Therefore, calculated ratios such as AIP and CRI I & II have been suggested to predict the risk of developing CVD. In the present study, the risk of developing CVD was found to be highest in the MONO and MOO groups using three ratios: AIP, CRI-I, and CRI-II. These are the subtypes of obesity that have MS. This finding is similar to other studies [13,14].

MS with a background of insulin resistance is associated with increased CV risk and mortality [9]. Insulin resistance develops prior to the development of type 2 diabetes mellitus, characterized by progressive beta-cell dysfunction. Increased abdominal obesity (visceral adiposity) is a significant risk factor for the development of MS as increased lipolysis occurs, leading to elevated free fatty acids and exacerbating insulin resistance, metabolic dysregulation, development of non-alcoholic fatty liver disease (NAFLD), and chronic kidney disease [15]. In Asian Indians, it was found that there was a high CV risk and high abdominal obesity (high visceral fat accumulation) even in individuals found to be in the normal BMI range. This could be attributed to the difference in the body composition between ethnic groups due to genetic polymorphisms that regulate fat storage and distribution, insulin sensitivity, and energy expenditure along with lifestyle, traditional diets of refined grains (increases postprandial glucose and insulin spikes), work culture, substance abuse, reduced physical activity, and unhealthy eating practices. Asian Indians are genetically predisposed to the "thin-fat" phenotype, characterized by lower skeletal muscle mass and accumulation of visceral adipose tissue (secretes pro-inflammatory cytokines (interleukin-6 (IL-6) and tumor necrosis factor-alpha (TNF-α)), which promote chronic low-grade inflammation and exacerbate endothelial dysfunction, a precursor to atherosclerosis and other CVDs) even at lower BMI [1].

In the present study, AIP was significantly correlated with BMI, VF%, and BF%. ROC analysis for comparing the AUC of the AIP, CRI-I, and CRI-II in predicting MS showed that AIP had the highest AUC (0.84) with an optimal cutoff point of 0.08 with a corresponding sensitivity of 78% and specificity of 81%. A cross-sectional study in Iran found that AIP was significantly correlated with BMI (r=0.33, p<0.001), WC, and physical activity [16]. Similarly, in a study in Taiwan comparing the predictive efficacy of AIP, CRI-I, and CRI-II, AIP had the highest AUC (0.845) with a cutoff point to predict MS of 0.04 with the corresponding sensitivity of 83.7% and specificity of 80.3% [14].

AIP is defined by the ratio of TG and HDL-C, and TG is directly associated with serum LDL-C levels. Studies have also found a relationship between AIP minute-dense LDL particles, the fraction of particles prone to oxidation, and, in turn, the production of foamy cells. HDL-C is an additional feature of AIP, which transports peripheral cholesterol to the liver and includes antioxidant enzymes, such as paraoxonase. Studies have verified these considerations by the direct association between AIP and carotid artery intima-media thickness, arterial stiffness, and coronary artery calcification [13,14,16,17].

The low sensitivity of CRI-I to detect subjects with MS could be due to the research project being conducted on a non-probability convenient sample, limiting the extrapolation of the results. Also, CRI-I (triglycerides/HDL) has a low sensitivity at a cutoff of >4.5814 to detect subjects with MS, or it is likely to fail to identify MS in a subject, probably because mean serum HDL levels are lower in an Indian population, resulting in higher mean CRI-I values in normal subjects also.

The study aimed to provide a deeper insight into how body fat distribution and obesity subtypes affect CV health. By utilizing ratios calculated from routine parameters for CV risk analysis, it moved beyond traditional BMI measurements for more accurate risk assessment with simplified clinical application.

However, this study has certain limitations as it was conducted only with a small number of subjects who presented to the hospital for the general health checkup, which lacks generalizability and may introduce selection bias, rendering the findings potentially not representative of the larger population. Being a study with a small sample size, there is an increased margin of error and reduced statistical power, making results less reliable. Outliers and biases can occur. Thus, the results are to be interpreted cautiously and validated by larger research in the future. The role of CRI I & II and the AIP need to be further assessed in larger general population studies comprising all age groups before being considered as a prospective screening marker for the development of MS. This study uses a BIA, which is less accurate than the gold standard test for body composition measurement, dual-energy X-ray absorptiometry (DEXA), because BIA has no radiation exposure hazards, unlike DEXA, which uses radiation and requires a specialized facility. Additionally, BIA is portable, affordable, user-friendly, non-invasive, and quick, allowing for easy measurements. It uses a safe, low-level electrical current.

The subjects of the study who were found to belong to the MOO, MONO, and MHO subtypes of obesity were directed to the General Medicine Outpatient Department with laboratory reports of their investigations and other details to be advised by the physician for appropriate counseling, lifestyle modifications, and institution of therapy if required.

## Conclusions

In conclusion, among the subtypes of obesity, the MOO and MONO groups exhibited high CV risk and, therefore, needed timely and more frequent screening and intervention. AIP showed a stronger association than CRI I & II with VF%, followed by FBS and BMI. AIP (AUC=0.841) had a good prediction capacity for the detection of MS compared with the CRI I & II. The awareness that BMI alone is insufficient to assess the risk of developing CV risks is now being understood, and various methods to expand and include the presence of other factors, such as MS, are being studied. Thus, classifying patients into subtypes of obesity and screening for CV risk using AIP and CRI I & II in general practice may facilitate healthcare providers to institute timely preventive measures to reduce the burden of non-communicable diseases.
